# Zanubrutinib as a salvage therapy for ibrutinib-resistant chronic lymphocytic leukemia: a case report

**DOI:** 10.3389/fonc.2025.1671124

**Published:** 2025-09-25

**Authors:** Zhenhui Lv, Ping Lu, Changhua Jing, Xuena Yi, Xueqiong Wu

**Affiliations:** Department of Hematology, Zibo Central Hospital, Zibo, Shandong, China

**Keywords:** ibrutinib, resistance, chronic lymphocytic leukemia, zanubrutinib, case report

## Abstract

**Objectives:**

This case report aims to evaluate the efficacy of zanubrutinib, a next-generation Bruton tyrosine kinase inhibitor (BTKi), in achieving durable disease control for patients with chronic lymphocytic leukemia/small lymphocytic lymphoma (CLL/SLL) who have experienced disease progression on ibrutinib, a first-generation BTKi.

**Methods:**

A patient with CLL/SLL who developed resistance to ibrutinib was treated with zanubrutinib. The patient’s clinical response, including disease progression, symptom control, and adverse effects, was monitored over time.

**Results:**

The patient demonstrated significant and durable disease control following the initiation of zanubrutinib therapy. The treatment was well-tolerated, with no severe adverse effects reported, suggesting that zanubrutinib may be an effective therapeutic option for ibrutinib-resistant CLL/SLL patients.

**Discussion:**

The findings from this case report suggest that zanubrutinib may provide a viable treatment option for patients with CLL/SLL who have progressed on ibrutinib. Further studies and clinical trials are warranted to confirm these observations and to explore the broader applicability of zanubrutinib in managing ibrutinib-resistant CLL/SLL.

## Introduction

The treatment landscape for CLL/SLL has changed significantly over the last decade owing to advancements in novel targeted therapies. The B-cell receptor (BCR) signaling pathway has emerged as a critical therapeutic target for CLL/SLL. Bruton’s tyrosine kinase (BTK) inhibitors are broadly categorized into covalent (irreversible) and non-covalent (reversible) agents. Covalent BTK inhibitors, such as ibrutinib, acalabrutinib, and zanubrutinib, bind irreversibly to the cysteine 481 (C481) residue in the BTK active site. In contrast, non-covalent inhibitors (e.g., pirtobrutinib) do not require binding to C481 and interact with BTK through reversible interactions. The primary mechanism of resistance to covalent BTK inhibitors is the acquisition of mutations in the BTK gene, most commonly the C481S mutation, which prevents the covalent binding of the drug. Secondary mutations in the downstream phospholipase C gamma 2 (PLCG2) gene can also confer resistance by sustaining B-cell receptor signaling independent of BTK activity (1).The first-generation BTKi of ibrutinib improves patient outcomes in CLL/SLL (2–4). The off-target effects of ibrutinib have been implicated in BTKi related adverse events(AEs). Zanubrutinib, a new-generation BTKi, was specifically engineered to optimize selectivity and maximize BTK occupancy (5, 6). Previous studies on the transformation of ibrutinib into zanubrutinib focused on intolerance to ibrutinib.Patients with ibrutinib intolerance were defined as those who were unable to continue the medication because of ibrutinib-related AEs (7). In the head-to-head ALPINE trial of zanubrutinib versus ibrutinib in patients with CLL, zanubrutinib showed a lower rate of AEs leading to death, discontinuation, dose reduction, and dose interruption (8). These data suggest that zanubrutinib may be a potential treatment option for ibrutinib intolerance. We report the case of a relapsed/refractory CLL patient who was resistant to ibrutinib and achieved new remission after transformation to zanubrutinib.

## Case presentation

A 74-year-old man with diabetes mellitus and hypertension was noted to have leukocytosis on a routine annual medical evaluation in June 2016. The patient’s personal and family history was noncontributory.His white blood cell count was 36.4 K/μL with lymphocytosis. Hemoglobin level and platelet count were normal. Flow cytometry (FCM) revealed a clonal population of small cells with CD19+, CD200+,CD20dim+, CD22dim+, CD5+, CD10−, CD38−, CD23+, FMC7−, kappa-, and lambda immunophenotypes. The patient had >1 poor prognostic variable, including unmutated immunoglobulin heavy chain variable region gene (IGHV 3–21 gene with a somatic hypermutation burden of 1.4%), del(17p) (76.5%), del(11q) (32%), and complex chromosomes(43,-Y,t(X;11;13;16) (q13;q23;p11;q13),-15,-18 (3)/46,XY (5)). At that time, a computed tomography (CT) scan of the neck, chest, and abdomen showed no enlarged lymph nodes or lymphoid organ such as the liver and spleen. The patient was diagnosed with CLL according to the World Health Organization (WHO) diagnostic criteria. The patient was advised to undergo clinical observation, given his early stage CLL.

He remained asymptomatic until December 2016 when he developed fatigue. He also started to have dark-colored urine, along with a decrease in hemoglobin levels, over a period of 2 weeks. The patient was evaluated at our hospital on December 25^th^, 2016. His white blood cell count was 8.8 K/μL, hemoglobin of 9.6 g/dL, and platelet count of 145 K/μL, respectively. The antinuclear antibody levels were normal. The direct anti-human ball test (Coombs test) was positive. Physical examination revealed no lymph nodes or hepatosplenomegaly. He received high-dose steroids for one week and sequential cyclophosphamide plus intravenous immunoglobulin to treat hemolysis due to hyperglycemia. However hemolysis was not stabilized. He felt his physical strength was declining, which was affecting his daily life.During the initial treatment, the patient exhibited hemolysis that was refractory to glucocortcoids, cyclophosphamide, and immunoglobulin. This led us to consider that the hemolysis was related to the underlying disease, and subsequently, we initiated treatment targeting the primary condition. The patient was then treated for CLL with rituximab (500 mg/m^2^ q4-6w) and chlorambucil (0.2 mg/kg.d*7d q4-6w) for eight cycles, which resulted in temporary stable control of hemolysis (2017.01-2018.02).

One month after the last rituximab, the patient developed pneumonia. His white blood cell count was 15 K/μL, with 80% lymphocytes and decreased hemoglobin and platelet counts. After aggressive anti-infection treatment, ibrutinib(420 mg qd) monotherapy was prescribed. The patient had a normal blood cell count that continued for 2.5 years (2018.02-2020.07). The patient was very satisfied with their quality of life.

The patient was readmitted for fatigue in July, 2020. At that time, his white blood cell count was 18.5 K/μL, with 89% lymphocytes, a decreased hemoglobin 9.3g/dL and a normal platelet count. The granulocyte was 5.2%, Coombs test was positive, and anti-nuclear antibody was negative. Computed tomography (CT) showed no obvious enlargement of the lymph nodes or hepatosplenomegaly. The progression of the primary disease was considered. A rituximab and high-dose methylprednisolone (R+HDMP) regimen consisting of rituximab 800 mg on day 0 and methylprednisolone 1500 mg on days 1–5 was given for 1 cycle. Hemoglobin rose briefly to 11.7 g/dL, but this did not last, and there was a gradual drop in platelet count. At the time, venetoclax and other non-covalent BTK inhibitors were not accessible in China as salvage therapy options, and the patient’s family declined chemotherapy. Based on the more sufficient and sustained inhibition of BTK by zanubrutinib compared to ibrutinib, we chose zanubrutinib (160mg bid) combined with rituximab(500 mg/m2 q4w) as the subsequent treatment on September 15^th^, 2020. One week after the treatment, the patient’s anemia quickly resolved. Then, rituximab plus zanubrutinib was used for another three cycles, followed by zanubrutinib monotherapy (160mg bid). The blood test results remained normal until December 2021.

The patient had no apparent cause for progressive hemoglobin or platelet reduction in December 2021. His white blood cell count was normal, but lymphocytes count was increased. The indirect bilirubin and lactate dehydrogenase levels were significantly increased. The Coombs test results were positive. CT showed several large focal liver lesions (greater than 34×21 mm), pancreatic body mass, splenomegaly, and extensive abdominal lymphadenopathy (19×14 mm). Tumor markers suggested a significant increase in a number of indicators such as CA199 (816 U/ml), CEA (17.6 ng/ml), CA125 (132 U/ml) and CA724 (17.6 U/ml). Cyclophosphamide and prednisone were administered to control hemolysis. Venetoclax (100 mg/day) was administered to control CLL for approximately two weeks. However, there was no improvement in the blood pattern. Simultaneously, ultrasound-guided liver biopsy revealed moderate to poorly differentiated adenocarcinoma. Second-generation sequencing of lymphoma (Peripheral Blood) revealed BTK p.C481S and TP53 mutations. We modified the treatment strategy to obinutuzumab combined with zanubrutinib. Obinutuzumab was administered intravenously until the patient died (on days 1, 8, and 15 of cycle 1, 1000 mg was administered. On day 1 of cycles 2-6, 1000 mg was administered) and concurrently with zanubrutinib 160 mg twice a day in a 28-day cycle. The patient’s blood pattern improved again (white blood cell count>3.5 K/μL, hemoglobin level > 10 g/dL, and platelet count > 80 K/μL) and six months later, the patient died of progressive liver damage.

Throughout the entire course of treatment, the patient’s main side effect was glucocorticoid-induced hyperglycemia. No BTK inhibitor-associated adverse events such as atrial fibrillation, bleeding, or hepatitis B reactivation were observed.

During the course of treatment with ibrutinib and subsequent zanubrutinib, the patient’s condition stabilized without significant adverse reactions. Furthermore, the ability to manage treatment orally at home greatly improved the patient’s quality of life, resulting in high satisfaction from both the patient and their family.

**Figure 1:**
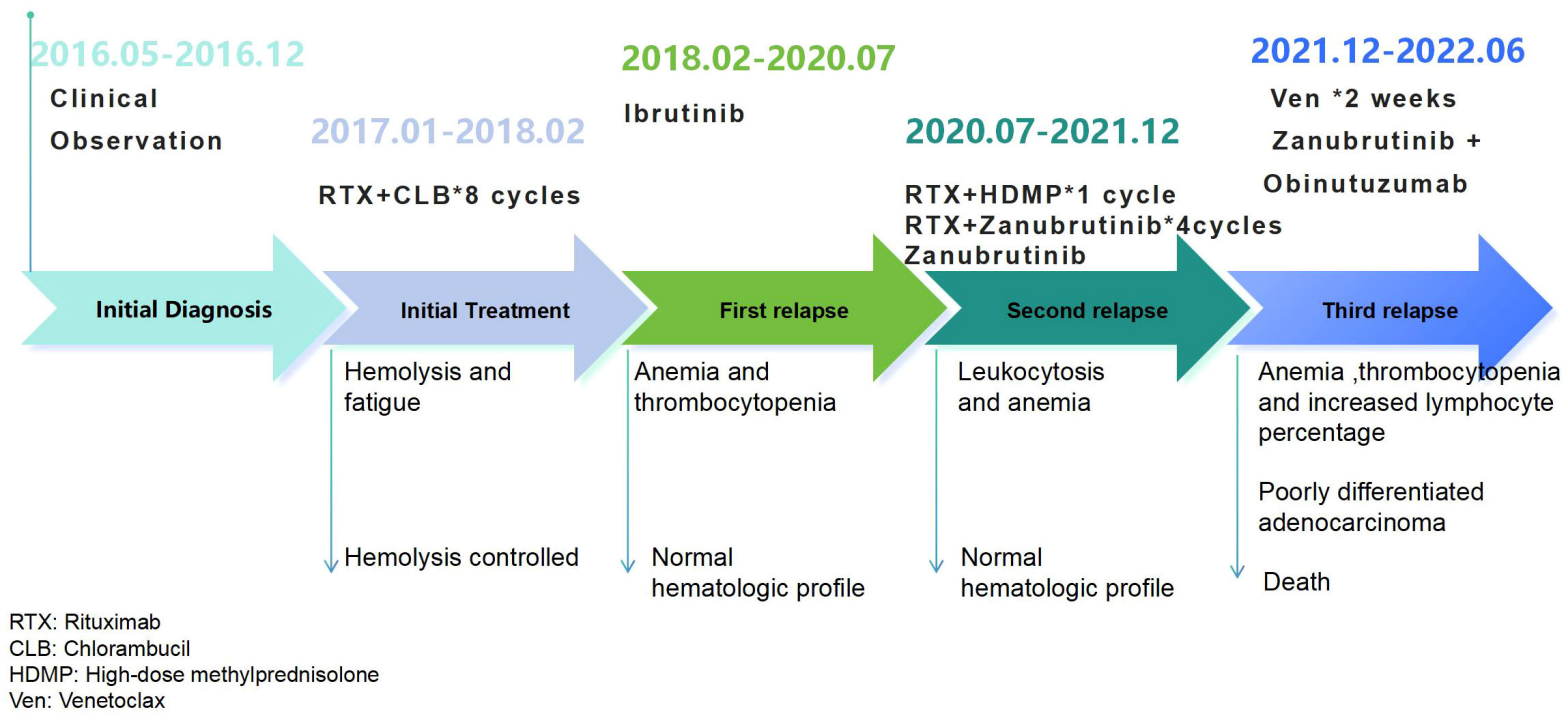
Schematic diagram of the patient's treatment course.

The patient’s treatment process is shown in **Figure 1**.

## Discussion

Treatment of CLL should be initiated in patients who meet any one of the following criteria: disease-related symptoms, low hemoglobin level, low platelet count, progressive or symptomatic spleen discomfort or massive splenomegaly, progressive or symptomatic lymphadenopathy or massive lymphadenopathy, rapidly increasing lymphocytosis, symptomatic or extra nodal involvement, and autoimmune complications not responding to steroids (9).

The optimal sequencing of targeted therapies in CLL remains a complex clinical challenge.As reviewed by Mouhssine et al., for the first-line treatment of low- and intermediate-risk CLL, both the BCL2 inhibitor venetoclax plus obinutuzumab and the second generation BTK inhibitors (BTKi), demonstrates favorable efficacy (10).Patients with 17p deletion, TP53 mutation, complex karyotype and an unmutated IGHV gene are considered to have high-risk diseases. These patients responded poorly to standard chemoimmunotherapy. Continuous therapy with acalabrutinib or zanubrutinib has shown clinically meaningful benefit in high-risk patients.The optimal treatment for relapsed/refractory CLL should be determined based on prior therapy and the duration of response to previous treatment, selecting either acalabrutinib/zanubrutinib or venetoclax combined with rituximab.Currently, novel treatment strategies to overcome resistance to pathway inhibitors include BTK degraders and immunotherapy with CAR-T or CAR-NK cells, though data on the efficacy of these therapeutic approaches remain insufficient (10).

Ibrutinib has shown substantial single drug efficacy in these patients and provide a chemotherapy free treatment option (3, 4). In the phase 3 RESONATE2 study, ibrutinib showed superior progression-free survival, overall survival, and overall response compared to chlorambucil in previously untreated older patients with CLL/SLL (11).

Although ibrutinib has excellent efficacy and general tolerability, AEs, such as bleeding, rash, and diarrhea, have been reported. These toxicities are related to ibrutinib discontinuation in both clinical trials and clinical practice settings (12). Compared to ibrutinib, zanubrutinib has shown greater selectivity for BTK with fewer off-target effects based on multiple *in vitro* enzymatic and cell-based assays. In a head-to-head phase 3 comparison of CLL, zanubrutinib treatment was associated with less toxicity, particularly cardiovascular toxicity, and a trend toward better response quality (13). The patient started ongoing ibrutinib and experienced progressive disease, and switching to zanubrutinib again showed efficacy, which was considered to be related to the sufficient inhibition of the BTK site by zanubrutinib, which needs to be confirmed in more clinical trials.

Zanubrutinib, ibrutinib, and other irreversible BTKi covalently bind cysteine 481 in the adenosine triphosphate–binding pocket of BTK (14). The affinity varied across these irreversible BTKi, depending on the specificity of the individual drug, for related and unrelated adenosine triphosphate–binding kinases that contain a sterically available cysteine at this position, including epidermal growth factor receptor (EGFR), interleukin-2–inducible T-cell kinase (ITK), and B-lymphocyte kinase et al. Anti-CD20 antibodies play a fundamental role in the treatment of patients with B-cell malignancies, with obinutuzumab having proven benefit (15, 16). The addition of obinutuzumab to ibrutinib may substantially improve depletion of CLL cells from the PB and BM (17). The relative sparing of ITK by zanubrutinib may result in less interference with the tumor-clearing mechanism of anti-CD20 antibody–induced antibody-dependent cytotoxicity, and then result in enhanced efficacy when combined with obinutuzumab. We hypothesized that obinutuzumab could be effectively used to treat BTKi-resistant CLL. After the occurrence the BTK p.C481S mutation, obinutuzumab was added with zanubrutinib, and the patient was stabilized again, which reflected this hypothesis.

With the use of BTKi, an increasing number of drug-resistant patients have emerged. The BTK p.C481S mutation was found to be resistant to BTKi (18).A limitation of this report is that genetic profiling for BTK (e.g., C481S) or PLCG2 mutations was not performed at the time of ibrutinib progression, prior to the commencement of zanubrutinib. One of the effective ways to treat these patients is to use the BCL2 inhibitor, venetoclax, which has shown durable remissions. The patient was intolerant of venetoclax. The investigation of ibrutinib resistance remains an ongoing clinical research topic.

## Conclusion

If disease progression occurs with ibrutinib monotherapy in CLL treatment, BTK p.C481S mutation should be screened. Conversion to zanubrutinib was considered in the absence of the mutation. When this mutation occurs, combination therapy with obinutuzumab should be considered.Due to the inherent limitations of a case report, the efficacy of zanubrutinib after ibrutinib resistance requires further validation in clinical practice.

## Data Availability

The datasets presented in this study can be found in online repositories. The names of the repository/repositories and accession number(s) can be found in the article/supplementary material.

## References

[1] Gruessner C Wiestner A Sun C. Resistance mechanisms and approach to chronic lymphocytic leukemia after BTK inhibitor therapy. Leuk Lymphoma. (2025) 66:1176–88. doi: 10.1080/10428194.2025.2466101, PMID: 39972943 PMC12221821

[2] Pan Z Scheerens H Li SJ Schultz BE Sprengeler PA Burrill LC. Discovery of selective irreversible inhibitors for Bruton’s tyrosine kinase. J ChemMedChem. (2007) 2:58–61. doi: 10.1002/cmdc.200600221, PMID: 17154430

[3] Burger JA Tedeschi A Barr PM Robak T Owen C Ghia P. RESONATE-2 investigators. Ibrutinib as initial therapy for patients with chronic lymphocytic leukemia. N Engl J Med. (2015) 373:2425–37. doi: 10.1056/NEJMoa1509388, PMID: 26639149 PMC4722809

[4] Byrd JC Brown JR O’Brien S Barrientos JC Kay NE Reddy NM RESONATE Investigators. Ibrutinib versus ofatumumab in previously treated chronic lymphoid leukemia. N Engl J Med. (2014) 371:213–23. doi: 10.1056/NEJMoa1400376, PMID: 24881631 PMC4134521

[5] Brown JR Robak T Ghia P Kahl BS Walker P Janowski W. Efficacy and safety of zanubrutinib in patients with treatment-naïve (TN) chronic lymphocytic leukemia (CLL) or small lymphocytic lymphoma (SLL) with del (17p): follow-up results from arm C of the SEQUOIA (BGB-3111-304) trial. Blood. (2020) 136:11–2. doi: 10.1182/blood-2020-134280

[6] Tam CS Trotman J Opat S Burger JA Cull G Gottlieb D. Phase 1 study of the selective BTK inhibitor zanubrutinib in B-cell Malignancies and safety and efficacy evaluation in CLL. Blood. (2019) 134:851–9. doi: 10.1182/blood.2019001160, PMID: 31340982 PMC6742923

[7] Guo Y Liu Y Hu N Yu D Zhou C Shi G. Discovery of zanubrutinib (BGB-3111), a novel, potent, and selective covalent inhibitor of bruton’s tyrosine kinase. J Med Chem. (2019) 62:7923–40. doi: 10.1021/acs.jmedchem.9b00687, PMID: 31381333

[8] Brown JR Eichhorst B Hillmen P Jurczak W Kaźmierczak M Lamanna N. Zanubrutinib or ibrutinib in relapsed or refractory chronic lymphocytic leukemia. N Engl J Med. (2023) 388:319–32. doi: 10.1056/NEJMoa2211582, PMID: 36511784

[9] Hallek M Cheson BD Catovsky D Caligaris-Cappio F Dighiero G Döhner H. iwCLL guidelines for diagnosis, indications for treatment, response assessment, and supportive management of CLL. Blood. (2018) 131:2745–60. doi: 10.1182/blood-2017-09-806398, PMID: 29540348

[10] Mouhssine S Maher N Kogila S Cerchione C Martinelli G Gaidano G. Current therapeutic sequencing in chronic lymphocytic leukemia. Hematol Rep. (2024) 16:270–82. doi: 10.3390/hematolrep16020027, PMID: 38804280 PMC11130833

[11] Barr PM Owen C Robak T Tedeschi A Bairey O Burger JA. Up to 8-year follow-up from RESONATE-2: first-line ibrutinib treatment for patients with chronic lymphocytic leukemia. Blood Adv. (2022) 6:3440–50. doi: 10.1182/bloodadvances.2021006434, PMID: 35377947 PMC9198904

[12] Kaur V Swami A. Ibrutinib in CLL: a focus on adverse events, resistance, and novel approaches beyond ibrutinib. Ann Hematol. (2017) 96:1175–84. doi: 10.1007/s00277-017-2973-2, PMID: 28342031

[13] Hillmen P Eichhorst B Brown JR Lamanna N O’Brien S Tam CS. CLL-115 First interim analysis of ALPINE : Results of a Phase 3 randomized study of zanubrutinib vs ibrutinib in patients with relapsed/refractory (R/R) chronic lymphocytic leukemia/small lymphocytic lymphoma (CLL/SLL). Clin Lymphoma Myeloma Leukemia. (2022) 22:s266.

[14] Herman SE Gordon AL Hertlein E Ramanunni A Zhang X Jaglowski S. Bruton tyrosine kinase represents a promising therapeutic target for treatment of chronic lymphocytic leukemia and is effectively targeted by PCI-32765. Blood. (2011) 117:6287–96. doi: 10.1182/blood-2011-01-328484, PMID: 21422473 PMC3122947

[15] Goede V Fischer K Busch R Engelke A Eichhorst B Wendtner CM. Obinutuzumab plus chlorambucil in patients with CLL and coexisting conditions. N Engl J Med. (2014) 370:1101–10. doi: 10.1056/NEJMoa1313984, PMID: 24401022

[16] Tam CS Quach H Nicol A Badoux X Rose H Prince HM. Zanubrutinib (BGB-3111) plus obinutuzumab in patients with chronic lymphocytic leukemia and follicular lymphoma. Blood Adv. (2020) 4:4802–11. doi: 10.1182/bloodadvances.2020002183, PMID: 33022066 PMC7556127

[17] Rawstron AC Hillmen P Maycock S Webster N Brock K Boucher RH. Ibrutinib and obinutuzumab in CLL: MRD responses sustained for several years with deepest MRD depletion in patients with> 1 year prior ibrutinib exposure. Blood. (2020) 136:27–8. doi: 10.1182/blood-2020-136990

[18] Woyach JA Furman RR Liu TM Ozer HG Zapatka M Ruppert AS. Resistance mechanisms for the Bruton’s tyrosine kinase inhibitor ibrutinib. N Engl J Med. (2014) 370:2286–94. doi: 10.1056/NEJMoa1400029, PMID: 24869598 PMC4144824

